# Nursing Workload and Optimal Staffing in Medical and Surgical Wards: A Time–Motion Study Using a Patient Classification System

**DOI:** 10.1155/jonm/3918250

**Published:** 2026-08-27

**Authors:** Muzelfe Biyik, Ensar Durmuş, Perihan Ersoy, Manar Aslan

**Affiliations:** ^1^ Department of Nursing, Kutahya Health Sciences University, Kutahya, Türkiye; ^2^ Ministry of Health of the Republic of Türkiye, Kutahya, Türkiye; ^3^ Kutahya Evliya Celebi Training and Research Hospital, Kutahya, Türkiye; ^4^ Department of Nursing, Trakya University, Edirne, Türkiye, trakya.edu.tr

**Keywords:** nurse staffing, nursing care, nursing staff, patient classification system, time and motion studies, workload

## Abstract

**Background:**

Evaluating the nursing workforce solely on the basis of nurse numbers leads to the neglect of critical workload determinants, such as indirect nursing activities and care intensity. This limitation may result in inaccuracies in workforce planning and pose risks to the quality of care. The combined use of time–motion analysis and patient classification systems enables a more comprehensive and objective assessment of nursing workload.

**Aim:**

This study aimed to analyse the workload of nurses working in the chest diseases and urology wards of a teaching and research hospital in Türkiye using time–motion analysis and to determine the optimal number of nurses (ONN) based on a patient classification system.

**Methods:**

This study employed a cross‐sectional, observational design based on time–motion analysis. Between June and July 2025, the workload of nurses working in the chest diseases and urology wards of a tertiary teaching hospital in Türkiye was evaluated. A total of 20 nurses were observed during morning and evening shifts over a 5‐week period. The ONN was calculated using three key indicators derived from the patient classification system: patient classification score, nursing intensity coefficient, and total care time.

**Findings:**

The calculated ONN was consistent with the current staffing levels in both wards. Higher care intensity (nursing intensity score: 9.44) in the chest diseases ward and patient volume (patient classification score: 55.75) in the urology ward emerged as the primary determinants of workload. Time–motion analysis showed that 22.8%–26.6% of nurses’ working time was allocated to direct care, 31.1%–32.9% to indirect care, and 34.6%–42.3% to personal tasks. Direct care time per patient was 12.60, 18.77, and 34.52 min for Group 1, 2, and 3 patients in the chest diseases ward and 11.18, 21.91, and 36.89 min, respectively, in the urology ward.

**Results:**

These findings indicate that nursing workload is shaped by different dynamics across clinical settings and that nurses allocate a limited proportion of their working time to direct patient care. The combined use of time–motion analysis and a patient classification system highlights the need for more accurate nurse staffing planning and workflow redesign to increase direct care time.

**Implications for Nursing Management:**

The findings provide nurse managers with an evidence‐based approach to assess nursing workload by integrating time–motion analysis, patient classification, and unit‐specific characteristics into staffing decisions. They can also use these findings to redesign workflows, improve documentation and medication‐preparation processes, and routinely monitor unit‐specific workload while preserving nurses’ essential rest and recovery activities.

## 1. Introduction

The global shortage of nurses represents a critical challenge for healthcare systems and poses a substantial threat to the continuity, safety, and quality of care. According to the OECD, the number of nurses per 1000 population in Türkiye is 2.6, which is considerably lower than the OECD average of 9.4 [1]. This structural shortage, combined with increasing ward demands and rising care complexity, places considerable pressure on the nursing workforce and creates significant risks for both nurse well‐being and patient safety [2–4]. A growing body of evidence indicates that excessive nursing workload is associated with adverse outcomes, including increased mortality, healthcare‐associated infections, medication errors, and nurse burnout [5–7]. From a nursing management perspective, understanding and accurately measuring workload is therefore essential for sustainable workforce planning and the delivery of safe, high‐quality care.

Within the framework of nursing job analysis, nurses’ activities are commonly classified into four categories: direct care, indirect care, ward‐related tasks, and personal time [8]. Direct care time, defined by the World Health Organisation (WHO) and the International Council of Nurses (ICN) as a key indicator of care quality, refers to activities involving direct interaction with patients [9]. These activities include assistance with activities of daily living, administration of treatments, communication, and psychosocial support. Indirect care comprises patient‐related activities that do not involve direct contact, such as documentation, handovers, interprofessional communication, and medication preparation [8].

Previous time‐and‐motion studies have demonstrated substantial variation in how nurses allocate their working time. Direct care has been reported to account for approximately 20%–54% of total working time (TWT), while indirect care constitutes 11%–40% [8, 10–15]. Ward‐related tasks—such as ward coordination, material management, supervision of students and newly employed staff, and participation in in‐ward education—have been reported to represent 1%–27% of nursing time [3, 15–17]. In addition, activities related to nurses’ personal needs, including meal and rest breaks, account for approximately 7%–16% of TWT [3, 8, 11]. These findings highlight that a substantial proportion of nurses’ working time is spent on activities other than direct patient care, which has important implications for staffing models and workflow design.

Determining the optimal number of nurses (ONN) is a central concern in nursing workforce management. However, there is no consensus on a single method for estimating staffing requirements, and existing approaches conceptualise nursing workload using different indicators and assumptions [14]. Moreover, although nursing activities and workload are known to vary across shifts, systematic evidence comparing workload distribution between shifts remains limited [11, 16]. This evidence gap constrains managers’ ability to make data‐driven decisions regarding staffing levels, workload distribution, and process redesign. Integrating time‐and‐motion analysis with patient classification systems may offer a more comprehensive and operationally useful approach to workload assessment and staffing planning.

Accordingly, the aim of this study was to analyse nurses’ workload across different shifts in an internal medicine ward (chest diseases) and a surgical ward (urology) using time‐and‐motion methodology and to determine the ONN based on a patient classification system.

### 1.1. Aim

This study aimed to analyse the workload of nurses working in the chest diseases and urology wards of a teaching and research hospital in Türkiye using time–motion analysis and to determine the ONN based on a patient classification system.

The study was guided by the following research questions:

• How much time do nurses in clinical wards spend on direct, indirect, and personal activities?
• Does the distribution of nurses’ workload differ by shift (morning–evening)?
• Does the level of patient care requirement change as patient type increases within clinical wards?
• What is the nursing intensity ratio in the clinical wards?
• What is the ONN required in the clinical wards?

## 2. Methods

### 2.1. Study Design

We conducted a cross‐sectional observational study between June 2025 and July 2025. In this article, we present the nursing observation data.

#### 2.1.1. Setting and Participants

This study was conducted in the chest diseases (32 beds) and urology (28 beds) wards of a teaching and research hospital in Türkiye with a total capacity of 520 beds. These wards were selected as they represent the busiest internal medicine and surgical wards within the hospital. Purposive sampling was used to include nurses who worked eight‐hour shifts in the selected departments and had at least 6 months of professional experience. Head nurses were excluded from the observations. Nurses were verbally informed about the study prior to data collection, and written informed consent was obtained on the day of observation. Participant characteristics, including age, length of professional experience, length of employment at the institution, and experience in the current department, were collected. Sample size was determined based on previously published time–motion studies and power analysis approaches [3, 17]. Assuming a medium effect size (*d* = 0.50), *α* = 0.05, and power (1−*β*) = 0.80, a minimum of 10 participants per ward was considered adequate. In time–motion studies, the primary unit of analysis is the total observation time rather than the number of participants. Accordingly, each nurse was observed for a full shift (approximately 8 h; ≈400–500 min), resulting in a total of approximately 160 h of observation across two wards. This observation duration is consistent with those reported in similar time–motion studies [3].

#### 2.1.2. Instruments

##### 2.1.2.1. Nursing Activities List

The Nursing Activities List was developed by the researchers and includes nursing activities performed in the wards, categorised as direct care, indirect care, ward‐related tasks, and personal tasks [16, 18]. Under the coordination of the hospital’s Health Care Services Manager, the final version of the list was established based on expert opinions from nurses working in the observed wards. The structured Nursing Activities List ensured that activities were classified using consistent criteria throughout the observation period, thereby enhancing inter‐observer consistency and reducing measurement error. Observation data were recorded using a structured data collection form that captured the ward name, shift during which the observation was conducted, type of nursing activity, duration of each activity, interruptions, and concurrent tasks.

##### 2.1.2.2. Rush Medicus Patient Classification System

The Rush Medicus Patient Classification System was used to determine patients’ dependency levels based on direct nursing care time [19]. The system consists of 29 items and categorises patients into four groups, with item scores ranging from 2 to 24. It classifies patients according to their care needs and the intensity of nursing interventions, thereby supporting the estimation of nursing workload and staffing requirements. As no Group 4 patients were present in the observed wards, this group was excluded from the analyses. Patient group levels were determined for each shift. These classifications were used to quantify patient‐related workload and were integrated with time–motion data to calculate workload scores. Based on the results of the time–motion analysis, patient classification scores were calculated for each patient using the average time required for each nursing activity.

#### 2.1.3. Pilot Study

Two experienced nurses working at the hospital who agreed to participate as observers were appointed as observers for the study [20]. Selecting nurses as observers ensured that the observations were conducted in a systematic and consistent manner, given their familiarity with clinical workflows and nursing activities. The observers received both theoretical and practical training from the principal investigator. The training covered the purpose and scope of the study, application of the observation method, use of the Nursing Activities List, operation of the stopwatch, and documentation procedures. During the observation period, observers were instructed to communicate with the observed nurses only when necessary.

As part of the pilot study, both observers simultaneously observed the same nurse for a three‐hour period. The pilot study aimed to assess inter‐observer reliability and to evaluate the applicability and clarity of the Nursing Activities List. Inter‐observer agreement was found to be high, with a correlation coefficient for activity types of *r*_*p*_ = 0.99.

### 2.2. Data Collection

On the days of observation, the researcher determined patients’ dependency levels using the Rush Medicus Patient Classification System. Classification of each patient required an average of 10 min. To account for weekly variability in patient profiles and nursing care processes, observations were conducted on different weekdays (Monday to Friday), with one day shift (08:00–16:00) and one evening shift (16:00–24:00) per week. Data collection was carried out through continuous observation throughout the entire shift. Observations commenced when the observed nurse began their shift and ended upon completion of their clinical duties. Data obtained from the observation forms were subsequently transferred to a digital format and stored securely in an electronic environment.

### 2.3. Data Analysis

Descriptive statistics, including frequencies, percentages, and means, were used to analyse participant characteristics, time‐and‐motion data, and patient classification system results. Statistical analyses were conducted using Microsoft Excel and IBM SPSS Statistics version 25.0, and time‐related variables were reported in hours and minutes.

Workload analysis was based on the nursing intensity calculation model developed by Ko and Park, which integrates patient classification data. A nurse’s TWT was calculated as the sum of direct nursing activities, indirect nursing activities, and personal time recorded during the observation period (Formula 1). The average direct nursing time per nursing intensity ward (DNTpNI) was calculated by considering the total direct nursing time (TDNT) in the clinic, the number of patients, and nursing intensity coefficients (Formula 2). Direct nursing time per patient (DNTpPt) was then calculated by multiplying DNTpNI by the weighting coefficient (WC) for the relevant patient group (Formula 3).

The care intensity for each patient group was calculated by dividing the total score obtained from the patient classification scale (b) by the daily average number of patients (a). WCs were determined to reflect differences in nursing intensity across patient groups. Using the patient classification score of ‘1’ in Group 1 as the reference value, WCs for Groups 2 and 3 were calculated. The contribution of each patient group to the total nursing workload (TNW) in the ward was determined using these nursing intensity WCs.

TDNT for each clinic was calculated as the sum of the products of the number of patients in each patient classification group and the corresponding DNTpPt (Formula 4). TNW was then obtained by adding indirect care time and personal time to the TDNT (Formula 5). As the proportions of indirect care and personal time were determined using clinic‐specific observational data, clinic‐specific coefficients were applied in the TNW calculations. Finally, the ONN was calculated by dividing the TNW by a nurse’s daily working hours (Formula 6). Nurses’ daily working hours were defined as 8 h, and a coefficient of 1.6 was applied as an adjustment factor to account for annual leave and public holidays.

## 3. Results

Ten nurses from each department were observed in the study. The mean age of nurses was 27.8 years in the chest diseases department and 31.1 years in the urology department. In both departments, the largest proportion of nurses had 1‐2 years of professional experience, employment at the institution, and experience within their current ward. Across the 10 observed shifts, the number of nurse‐days was higher in the chest diseases department, whereas the number of patient‐days was higher in the urology department (Table 1).

**TABLE 1:** Demographic and work‐related characteristics of nurses by ward.

Variables	Chest disease ward (*n* = 10)	Urology ward (*n* = 10)
Age (mean ± SD (range))	27.8 ± 2.9 (24–31)	31.1 ± 6.7 (24–40)
Years of professional experience (1‐2 years)^∗^ *n* (%)	3 (30.0)	4 (40.0)
Length of employment in the current institution (1‐2 years)^∗^ *n* (%)	4 (40.0)	5 (50.0)
Years of experience in the current ward (1‐2 years)^∗^ *n* (%)	10 (100.0)	10 (100.0)
Nurse‐days (10‐day period)	40	35
Patient‐days (10‐day period)	229	250

^∗^Most common category: 1–2 years.

The mean daily number of patients was 22.9 in the chest diseases ward and 25.0 in the urology ward. In the chest diseases ward, the patient classification score was 97.6, and the nursing intensity score was 9.44. In contrast, the urology ward had a patient classification score of 55.75 and a nursing intensity score of 1.42 (Table 2).

**TABLE 2:** Comparison of nursing intensity by nursing wards.

Nursing ward	Patient classification	Average number of patients per day	Patient classification score	Patient classification score per patient	Weighting coefficient	Nursing intensity weighting coefficient	Average number of nurses per day	Number intensity score
		*a*	*b*	*c = b/a*	*d*	*axd*		
Chest diseases	Group 1	5.8	432.573	59.06	1	5.8	4	9.44
	Group 2	11.9	1050.291	88.26	1.49	17.73		
	Group 3	5.2	842.295	161.98	2.74	14.25		
	Total	22.9	2235.16	97.6	1.65	37.79		

Urology	Group 1	11.5	399.900	34.77	1	11.5	3.5	11.42
	Group 2	12.0	820.265	68.35	1.96	23.52		
	Group 3	1.5	173.479	115.65	3.3	4.95		
	Total	25	1393.64	55.75	1.6	40		

In the chest diseases ward, 26.6% of nurses’ working time was allocated to direct care and 32.9% to indirect care, while personal time accounted for 34.6% of the TWT. In the urology ward, the corresponding proportions were 22.8% for direct care, 31.1% for indirect care, and 42.3% for personal time (Table 3).

**TABLE 3:** Total working hours, nondirect nursing time and leisure time.

			Chest diseases									Urology								
			Day			Evening shift			Total			Day			Evening shift			Total		
Number of nurses (person)		Subtasks	Daily average (min)	Average time per person (min)	%	Daily average (min)	Average time per person (min)	%	Daily average (min)	Average time per person (min)	%	Daily average (min)	Average time per person (min)	%	Daily average (min)	Average time per person (min)	%	Average time per person (min)	Daily average (min)	%
Task																				
DNT		Total duration	667.34	166.53	28.8	584.12	146.03	24.5	1251.46	312.56	26.6	650.50	185.57	27.1	427.52	122.14	18.3	1078.20	308.12	22.8
		Administration of medication	77.10	19.7	3.3	141.19	35.19	5.9	218.29	54.37	4.6	96.19	27.31	4.0	150.24	42.58	6.4	246.43	70.29	5.2
		Interventions of care	159.18	39.49	6.9	287.59	71.59	12.1	447.17	111.49	9.5	218.31	62.26	9.1	219.42	62.46	9.4	438.13	125.12	9.3
		Patient transfer	276.58	69.14	11.9	57.20	14.20	2.4	334.18	83.34	7.1	142.55	40.50	6.0	38.25	10.58	1.6	181.20	51.48	3.8
		Patient admission and discharge	154.08	38.32	6.6	97.34	24.23	4.1	251.42	62.55	5.4	193.05	55.10	8.0	19.21	5.31	0.8	212.26	60.41	4.5

NDNT	1	Total duration	694.46	173.41	30.0	850.30	212.37	35.7	1545.16	386.19	32.9	782.35	223.35	32.6	691.04	197.26	29.6	1473.39	421.02	31.1
		Preparation for care interventions	93.09	23.17	4.0	80.42	20.10	3.4	173.51	43.27	3.7	91.18	26.05	3.8	53.20	15.14	2.3	144.38	41.19	3.1
		Medication preparation	105.31	26.22	4.6	347.30	86.52	14.6	453.01	113.15	9.6	142.31	40.43	5.9	153.45	43.55	6.6	296.16	84.38	6.3
		Documentation	313.47	78.26	13.5	238.19	59.34	10.0	552.06	138.01	11.7	387.20	110.40	16.1	335.20	95.48	14.4	722.40	206.28	15.3
		Communication	182.19	45.34	7.9	183.59	45.59	7.7	366.18	91.34	7.8	158.20	45.14	6.6	148.39	42.28	6.4	306.59	87.42	6.5
	2	Total duration	156.40	39.11	6.8	119.41	29.55	5.0	276.28	69.07	5.9	104.11	29.46	4.3	76.17	21.47	3.3	180.28	51.33	3.8
		Ward organisation	34.46	8.41	1.5	28.25	7.06	1.2	63.11	15.47	1.3	45.16	12.56	1.9	11.46	3.21	0.5	57.02	16.17	1.2
		Waiting	24.34	6.08	1.1	53.14	13.18	2.2	77.48	19.27	1.7	30.01	8.34	1.3	41.55	11.58	1.8	71.56	20.33	1.5
		Obtaining medicines from the pharmacy	97.27	24.21	4.2	38.02	9.30	1.6	135.29	33.52	2.9	28.54	8.15	1.2	22.36	6.27	1.0	51.30	14.42	1.1

PT		Total duration	799.25	199.51	34.5	826.26	206.36	34.7	1625.51	406.27	34.6	862.05	246.18	35.9	1140.04	325.44	48.8	2002.09	572.02	42.3
		Sitting at the desk	286.01	71.30	12.3	163.27	40.51	6.9	449.28	112.22	9.6	341.32	97.34	14.2	249.26	71.16	10.7	590.58	168.50	12.5
		Phone call	175.15	43.48	7.6	2.35	0.38	0.1	177.50	44.27	3.8	13.27	3.50	0.6	8.02	2.17	0.3	21.29	6.08	0.5
		Meal/tea time	231.02	57.45	10.0	255.34	63.53	10.7	486.36	121.39	10.4	323.17	92.22	13.5	592.18	169.13	25.4	915.35	261.35	19.3
		Restroom/prayer	107.07	26.46	4.6	404.50	101.12	17.0	511.57	127.59	10.9	183.49	52.31	7.7	290.18	82.56	12.4	474.07	135.27	10.0

Total			2318.32	579.38	100	2380.49	595.12	100	4699.21	1174.50	100	2399.41	685.37	100	2335.17	667.13	100	4734.58	1352.50	100

Average number of nurses per shift			4.0									3.5								

*Note:* Total working time (TWT) was calculated as the sum of direct nursing time (DNT), nondirect nursing time (NDNT), and personal time (PT), i.e., TWT = DNT + NDNT + PT.

Abbreviations: DNT, direct nursing time; NDNT, nondirect nursing time; PT, personal time; TWT, total working time.

In the chest diseases ward, the direct nursing care time per patient calculated according to patient classification groups was 12.6 min for Group 1 patients, 18.77 min for Group 2 patients, and 34.52 min for Group 3 patients. In the urology ward, the corresponding direct nursing care times were calculated as 11.18, 21.91, and 36.89 min for Groups 1, 2, and 3 patients, respectively (Table 4).

**TABLE 4:** Direct nursing time per patient in the nursing wards.

Nursing wards	Patient classification	WC	DNTpNI	DNTpPt (min)
Chest diseases	Group 1	1.00	12.60	12.60
	Group 2	1.49	12.60	18.77
	Group 3	2.74	12.60	34.52

Urology	Group 1	1.00	11.18	11.18
	Group 2	1.96	11.18	21.91
	Group 3	3.30	11.18	36.89

*Note:* DNTpNI, direct nursing time per nursing intensity weighting coefficient; DNTpPt, direct nursing time per patient. DNTpNI was calculated by dividing the total direct nursing time by the weighted number of patients. DNTpPt was then calculated by multiplying DNTpNI by the corresponding weighting coefficient (WC).

Abbreviation: WC, weighting coefficient.

In the chest diseases ward, the actual number of nurses was 11, while the ONN was calculated as 10.51. Similarly, in the urology ward, where 11 nurses were employed, the ONN was calculated as 10.86. These findings indicate that the current staffing levels in both wards were close to the optimal level (Table 5).

**TABLE 5:** Calculation of the optimal number of nurses in nursing wards.

Parameter	Chest diseases	Urology
TNW (h)	52.55 h	54.33 h
Formula 5: TNW = TDNT + TNDNT		
TDNT (h)	20.85 h	17.96 h
Formula 4: (*m*_1_ × DNTpPt_1_) + (*m*_2_ × DNTpPt_2_) + (*m*_3_ × DNTpPt_3_)		
TNDNT	31.70 h	36.36 h
Formula for chest diseases: TDNT × 1.626		
Formula for urology: TDNT × 2.305		
ONN (persons)	11 (10.51)	11 (10.86)
Available number of nurses in each ward (persons)	11	11
Number of nurses requiring additional employment (persons)	0	0

*Note:* Total nursing workload (TNW) was calculated by combining direct and nondirect nursing time, and the optimal number of nurses (ONN) was estimated based on daily working hours with an adjustment coefficient.

Abbreviations: ONN, optimal number of nurses; TDNT, total direct nursing time; TNDNT, total nondirect nursing time; TNW, total nursing workload.

## 4. Discussion

This study demonstrated the objective and comparable nature of nursing workload in the chest disease and urology wards by integrating time–motion analysis with a patient classification system. Although the ONN in both wards was consistent with existing staffing levels, nurses were found to spend a substantial proportion of their working time on indirect care and personal activities. This finding indicates a need for workflow reorganisation to increase the proportion of time allocated to direct patient care. In addition, care intensity emerged as the primary determinant of workload in the chest diseases ward, whereas high patient volume was the main driver of workload in the urology ward. These results highlight the importance of incorporating both clinical characteristics and direct care time into nursing workforce planning.

In this study, the proportion of time devoted to direct care was comparable to the 20%–25% range reported in some previous studies [12, 14]. However, the literature indicates considerable variability in direct care ratios, ranging from 30% to 54% [8, 11, 13, 15, 21]. One key reason for these discrepancies is the lack of consistency in how direct care is defined across studies. In the present study, direct care was limited to face‐to‐face interactions between nurses and patients, whereas other studies have adopted a broader conceptualisation under the framework of ‘value‐adding care’. This approach typically includes direct care, indirect care, and documentation activities [8].

Nevertheless, the finding that approximately one‐quarter of nurses’ TWT was allocated to direct care suggests that opportunities for nurse–patient interaction may be limited. The relatively high proportion of time devoted to indirect care and personal activities may further constrain the time available for direct care delivery [22, 23]. This pattern is likely attributable, in part, to the extensive time required for paper‐based documentation and the labour‐intensive nature of medication preparation processes.

In the present study, indirect care accounted for 32.9% of working time in the chest diseases ward and 31.1% in the urology ward. Previous research has reported wide variability in indirect care proportions, ranging from 7.2% to 79.5%. In Turkey, however, indirect care time is generally reported within a narrower range, typically between 16.6% and 24.5% [11–13, 16, 17, 24]. Accordingly, the indirect care ratios observed in this study exceed those reported in the national literature and approach the upper range of values documented in international studies. Such high levels of indirect care pose a potential risk by limiting the time nurses can devote to direct patient care and by potentially disrupting the continuity and quality of clinical care processes.

A substantial proportion of working time was allocated to documentation and medication management processes, which constitute key components of indirect care. In the observed wards, the predominantly paper‐based nature of documentation emerged as one of the main factors contributing to the prolonged duration of these activities. The literature consistently indicates that electronic health information systems enhance nursing workflow by reducing documentation time and enabling nurses to allocate more time to direct patient care [22, 23, 25]. Accordingly, the reliance on paper‐based systems may partly explain the high indirect care ratios observed in the present study. From this perspective, the implementation of electronic health record systems appears to be a critical requirement for improving time management and care efficiency [26].

Medication management, another major component of indirect care, has been reported in the literature to account for between 11% and 28.7% of nurses’ working time [11, 15, 16]. This wide variation has been attributed primarily to methodological differences in how nursing activities are categorised across studies [27]. In the present study, medication administration was classified as direct care, medication preparation as indirect care, and medication‐related recording as documentation. This classification approach may have resulted in medication management accounting for a smaller proportion of total time compared with some previous studies. Nevertheless, the findings suggest that time loss is particularly pronounced in documentation and medication preparation processes, highlighting the need for improvements in nursing workflows in these areas. Therefore, even when staffing levels are optimal, adjustments in task allocation, process standardisation, and information technology infrastructure remain necessary.

This study also found that nurses spent more than one‐third of their working time on personal activities. This proportion exceeds that reported in many time–motion studies [3, 8, 10, 16, 17, 28], although it is consistent with the findings reported by Aslan et al. [29]. The relatively high proportion of personal time may reflect both essential recovery activities and structural factors such as high workload, frequent interruptions, role ambiguity, and systemic constraints within the work environment. Turkmen and Uslu [28] suggest that personal time may function as a coping strategy for nurses experiencing chronic fatigue and burnout. It should be noted that the personal time category in this study included essential activities such as meal and rest breaks, restroom/prayer, and desk‐based activities. Therefore, a relatively high proportion of personal time should not necessarily be interpreted as inefficient or inappropriate time use. Rather, it highlights the importance of distinguishing essential recovery activities from avoidable workflow inefficiencies when evaluating nursing workload and planning organisational improvements. Taken together, these findings point to a significant organisational challenge that hinders the achievement of recommended direct and indirect care ratios under current working conditions.

It should also be acknowledged that some aspects of the observed workload distribution may reflect organisational characteristics specific to the study setting. For example, the relatively high proportions of indirect care and personal time may have been influenced by local documentation practices, workflow organisation, staffing structures, and institutional culture. Another contextual factor that may have influenced the findings is the relatively limited clinical experience of the participating nurses. In both wards, the most common category for professional experience, institutional tenure, and ward experience was 1–2 years. Less experienced nurses may require more time for documentation, medication preparation, communication, and adaptation to clinical workflows [11, 15], which could have contributed to the relatively high proportions of indirect care and personal time observed in this study. Therefore, although the methodology used in this study provides an objective framework for workload assessment, the exact proportions of time allocated to different activity categories should be interpreted within the context of the participating hospital rather than assumed to represent all clinical settings.

Although patients’ care dependency was higher in the chest diseases ward, it is noteworthy that the workload per nurse was greater in the urology ward. This finding suggests that workload in the chest diseases ward is primarily influenced by care intensity, whereas in the urology ward it is driven by a higher patient volume. The observation that a substantial proportion of direct care time in the urology ward was devoted to admission and discharge procedures further supports this interpretation. The literature indicates that nursing workload varies considerably according to clinical characteristics and organisational factors [14]. Similarly, Van Bogaert et al. [30] reported that even when staffing levels are adequate, a high burden of indirect tasks may restrict the time available for direct patient care.

The present findings demonstrate that direct care time was limited due to workflow organisation and task distribution, highlighting the need to incorporate both clinical characteristics and direct care time into workload planning. Moreover, direct care time increased markedly with higher patient classification levels in both wards. In particular, direct care time for Group 3 patients was higher in the urology ward than in the chest diseases ward. This finding underscores the necessity of clinic‐specific nursing workforce planning for patient groups with high care demands. It is consistent with the results reported by Lv et al. [31] and supports the view that nursing workforce planning should be tailored to patient classification groups rather than based solely on overall patient numbers.

In this study, the ONN in both wards was found to be consistent with the existing staffing levels. However, the substantial proportion of time allocated to indirect care and personal activities suggests that nursing workforce planning may be inefficient if it is based only on staffing numbers. By integrating time–motion analysis with a patient classification system, this study enabled a standardised, objective, and comparable assessment of nursing workload across clinical settings. Therefore, nursing workforce planning may benefit from a multifaceted approach that incorporates not only patient numbers and care intensity but also nurses’ actual use of time in clinical practice.

### 4.1. Implications for Nursing Management

The findings of this study have practical implications for nursing management and workforce planning. Relying solely on nurse‐to‐patient ratios may be insufficient for accurately assessing nursing workload. Nurse managers can use these findings to support evidence‐based staffing assessments by integrating patient classification, direct care time, and unit‐specific characteristics into staffing decisions. They can also use the findings to redesign workflows, improve documentation and medication‐preparation processes, and routinely monitor unit‐specific workload. Such strategies should focus on reducing avoidable inefficiencies while preserving nurses’ essential rest and recovery activities, thereby increasing the time available for direct patient care.

## 5. Conclusion

This study provides an objective assessment of nursing workload by integrating time–motion analysis with a patient classification system. Although the number of nurses in both clinics was found to be consistent with the calculated ONNs, a considerable proportion of nurses’ working time was allocated to indirect care and personal activities. In the chest diseases ward, workload was mainly influenced by the intensity of care, whereas in the urology ward, it was driven largely by patient volume. These findings indicate that effective workforce planning should not focus solely on staffing numbers but also consider how nurses’ time is distributed across different care activities and clinical contexts.

### 5.1. Strengths and Limitations

This study was conducted in a single hospital and included only two clinical wards, which limits the generalisability of the findings. Although the number of observed nurses was limited, this is typical for time–motion studies. The extensive observation time supports the robustness of the data; however, the findings should be interpreted with caution regarding generalisability. In addition, as observations were restricted to day and evening shifts, nursing workload and task distribution during night shifts could not be evaluated. The relatively short length of nurses’ professional and ward‐specific experience, together with organisational characteristics unique to the study hospital (e.g., documentation practices, workflow arrangements, and institutional culture), may also have influenced the observed workload patterns. Therefore, caution is warranted when generalising these findings to other clinical settings. Furthermore, the inability to separately record the duration of secondary activities when nurses performed multiple tasks simultaneously may have resulted in an incomplete reflection of overall nursing workload. Nurses’ behaviour may also have been influenced by being observed, potentially leading to increased productivity due to the Hawthorne effect; although observers were trained and interaction with participants was minimised, this effect may have partially affected the results. Despite these limitations, a major strength of this study lies in the use of time–motion analysis in combination with a patient classification system to evaluate nursing workload in a standardised and objective manner. Conducting the study in two different clinical wards enabled the identification of workload differences based on clinic type, and the simultaneous calculation of workload scores and optimal nurse staffing levels provided a comprehensive and practice‐oriented framework for nursing workforce planning. Future studies should replicate this methodology across different hospital types and clinical specialities to evaluate the transferability of the findings.

## Author Contributions

Conceptualization: Muzelfe Biyik, Ensar Durmuş, Perihan Ersoy and Manar Aslan.

Methodology and formal analysis: Muzelfe Biyik and Manar Aslan.

Data collection: Muzelfe Biyik and Perihan Ersoy.

Writing–original draft: Muzelfe Biyik.

Writing–review and editing: Ensar Durmuş, Perihan Ersoy and Manar Aslan.

## Funding

This research received no external funding.

## Disclosure

All scientific content, study design, data analysis, interpretation of the findings and final manuscript revisions were performed and approved by the authors.

## Ethics Statement

Ethical approval for this study was obtained from the Kütahya Health Sciences University Ethics Committee (approval no: 46; Date: May 2025). The study was conducted in accordance with the principles of the Declaration of Helsinki. Written informed consent was obtained from all participants prior to data collection.

## Conflicts of Interest

The authors declare no conflicts of interest.

## Supporting Information

Additional supporting information can be found online in the Supporting Information section.

## Supporting information


**Supporting Information** STROBE‐checklist JNM.

## Data Availability

The datasets generated and/or analysed during the current study are available from the corresponding author on reasonable request.

## References

[1] OECD , OECD Health Statistics 2023, 2023, OECD Publishing.

[2] Nantsupawat A. , Nantsupawat R. , Kulnaviktikul W. , and McHugh M. D. , Relationship Between Nurse Staffing Levels and Nurse Outcomes in Community Hospitals, Thailand, Nursing and Health Sciences. (2015) 17, no. 1, 112–118, 10.1111/nhs.12140. 24698300

[3] Al-Moteri M. , Alzahrani A. A. , Althobiti E. S. et al., The Road to Developing Standard Time for Efficient Nursing Care: A Time and Motion Analysis, Healthcare. (2023) 11, no. 15, 10.3390/healthcare11152216. PMC10418769 37570456

[4] Lasater K. B. , Aiken L. H. , Sloane D. et al., Patient Outcomes and Cost Savings Associated With Hospital Safe Nurse Staffing Legislation: An Observational Study, BMJ Open. (2021) 11, no. 12, 10.1136/bmjopen-2021-052899. PMC8655582 34880022

[5] Witczak I. , Rypicz Ł. , Karniej P. , Młynarska A. , Kubielas G. , and Uchmanowicz I. , Rationing of Nursing Care and Patient Safety, Frontiers in Psychology. (2021) 12, 10.3389/fpsyg.2021.676970. PMC8458807 34566757

[6] McHugh M. D. , Aiken L. H. , Windsor C. , Douglas C. , and Yates P. , Case for Hospital Nurse-to-Patient Ratio Legislation in Queensland, Australia: An Observational Study, BMJ Open. (2020) 10, no. 9, 10.1136/bmjopen-2019-036264. PMC7476482 32895270

[7] Diehl E. , Rieger S. , Letzel S. et al., The Relationship Between Workload and Burnout Among Nurses: The Buffering Role of Personal, Social and Organisational Resources, PLoS One. (2021) 16, no. 1, 10.1371/journal.pone.0245798. PMC7822247 33481918

[8] Antinaho T. , Kivinen T. , Turunen H. , and Partanen P. , Nurses’ Working Time Use–How Value Adding is It?, Journal of Nursing Management. (2015) 23, no. 8, 1094–1105, 10.1111/jonm.12258. 25280350

[9] World Health Organization (WHO) & International Council of Nurses (ICN) , Strategic Directions for Nursing and Midwifery 2022–2025, 2022, World Health Organization.

[10] Farquharson B. , Bell C. , Johnston D. et al., Frequency of Nursing Tasks in Medical and Surgical Wards, Journal of Nursing Management. (2013) 21, no. 6, 860–866, 10.1111/jonm.12110. 23924377

[11] Michel O. , Garcia Manjon A. J. , Pasquier J. , and Ortoleva Bucher C. , How do Nurses Spend Their Time? A Time and Motion Analysis of Nursing Activities in an Internal Medicine Unit, Journal of Advanced Nursing. (2021) 77, no. 11, 4459–4470, 10.1111/jan.14935. 34133039 PMC8518809

[12] Abt M. , Lequin P. , Bobo M. L. , Vispo Cid Perrottet T. , Pasquier J. , and Ortoleva Bucher C. , The Scope of Nursing Practice in a Psychiatric Unit: A Time and Motion Study, Journal of Psychiatric and Mental Health Nursing. (2022) 29, no. 2, 297–306, 10.1111/jpm.12790. 34310817 PMC9290684

[13] Caron I. , Gélinas C. , Boileau J. , Frunchak V. , Casey A. , and Hurst K. , Initial Testing of the Use of the Safer Nursing Care Tool in a Canadian Acute Care Context, Journal of Nursing Management. (2021) 29, no. 6, 1801–1808, 10.1111/jonm.13300. 33650195 PMC8519130

[14] Ko Y. and Park B. , Calculating the Optimal Number of Nurses Based on Nursing Intensity by Patient Classification Groups in General Units in South Korea: A Cross-Sectional Study, Nursing Open. (2023) 10, no. 6, 3982–3991, 10.1002/nop2.1644. 36852629 PMC10170926

[15] Kramer S. , Raymond M. J. , Hunter P. et al., Understanding the Workflow of Nurses in Acute and Subacute Medical Wards: A Time and Motion Study, Journal of Clinical Nursing. (2023) 32, no. 21–22, 7773–7782, 10.1111/jocn.16835. 37489643

[16] Ekici D. and Gurkay E. , Nursing Time Allocation: A Work Sampling Survey in a Turkish Private Hospital, International Journal of Hospital Research. (2016) 5, no. 2, 58–63.

[17] Koç Z. and Alpar S. E. , The Effect of Lean Hospital Practices on Nurses’ Direct Care Activities: A Time and Motion Study, Journal of Evaluation in Clinical Practice. (2025) 31, no. 1, 10.1111/jep.14278. 39699068

[18] Republic of Türkiye Ministry of Health , Nursing Regulation, 2010, Official Gazette of the Republic of Türkiye, https://www.resmigazete.gov.tr.

[19] Velioglu P. , Bildirici Z. , Management Support Techniques, Health Institutions Management, 1993, Anadolu University.

[20] Lopetegui M. , Yen P. Y. , Lai A. , Jeffries J. , Embi P. , and Payne P. , Time Motion Studies in Healthcare: What are We Talking About?, Journal of Biomedical Informatics. (2014) 49, 292–299, 10.1016/j.jbi.2014.02.017. 24607863 PMC4058370

[21] Seong J. , Cho S. H. , Yoon H. J. , Sim W. H. , and Kim M. S. , Comparison of Nurse Work Hours and Nursing Activities Between High- and Low-staffed General Wards: A Cross-Sectional Study, Nursing Open. (2024) 11, no. 12, 10.1002/nop2.70109. PMC11623342 39642138

[22] Yen P. Y. , Kellye M. , Lopetegui M. et al., Nurses’ Time Allocation and Multitasking of Nursing Activities: A Time Motion Study, AMIA Annual Symposium Proceedings. (2018) 2018, 1137–1146. 30815156 PMC6371290

[23] Moore E. C. , Tolley C. L. , Bates D. W. , and Slight S. P. , A Systematic Review of the Impact of Health Information Technology on Nurses’ Time, Journal of the American Medical Informatics Association. (2020) 27, no. 5, 798–807, 10.1093/jamia/ocz231. 32159770 PMC7309250

[24] Ko Y. and Park B. , Calculating the Optimal Number of Nurses Based on Nursing Intensity by Patient Classification Groups in General Units in South Korea: A Cross-Sectional Study, Nursing Open. (2023) 10, no. 6, 3982–3991, 10.1002/nop2.1657. 36852629 PMC10170926

[25] Wong D. , Bonnici T. , Knight J. , Gerry S. , Turton J. , and Watkinson P. , A Ward-Based Time Study of Paper and Electronic Documentation for Recording Vital Sign Observations, Journal of the American Medical Informatics Association. (2017) 24, no. 4, 717–721, 10.1093/jamia/ocw186. 28339626 PMC7651906

[26] Peršolja M. , Intensity of Nursing Work in a Primary Healthcare Center: an Observational Study, International Journal of Nursing Science. (2024) 11, no. 5, 536–543, 10.1016/j.ijnss.2024.10.007. PMC11650679 39698135

[27] Griffiths P. , Saville C. , Ball J. , Culliford D. , Pattison N. , and Monks T. , Performance of the Safer Nursing Care Tool to Measure Nurse Staffing Requirements in Acute Hospitals: A Multicentre Observational Study, BMJ Open. (2020) 10, no. 5, 10.1136/bmjopen-2019-035828. PMC7232629 32414828

[28] Turkmen E. and Uslu A. , Evaluation of Indirect Nursing Care Practices in a Private Hospital, Florence Nightingale Journal of Nursing. (2011) 19, no. 2, 60–67.

[29] Aslan M. , Karaaslan A. , Yıldız A. , Doğan F. , and Evirgen H. , Workload of Nurses and Care Left Undone: Do We Really Care Enough?, International Journal of Caring Sciences. (2016) 9, no. 2.

[30] Van Bogaert P. , van Heusden D. , Timmermans O. , and Franck E. , Nurse Work Engagement Impacts Job Outcome and Nurse-Assessed Quality of Care, Frontiers in Psychology. (2014) 5. 10.3389/fpsyg.2014.01261 PMC4230203 25431563

[31] Lv C. , Gong Y. H. , Wang X. H. , An J. , Wang Q. , Han J. , and Chen X. F. , Correlation Between Diagnosis-Related Group Weights and Nursing Time in the Cardiology Department: Cross-Sectional Study, JMIR Medical Informatics. (2025) 13, 10.2196/65549. PMC11896087 40036488

